# Clinical and Physiologic Factors Associated With Mode of Death in Pediatric Severe TBI

**DOI:** 10.3389/fped.2021.793008

**Published:** 2021-12-13

**Authors:** Talia D. Baird, Michael R. Miller, Saoirse Cameron, Douglas D. Fraser, Janice A. Tijssen

**Affiliations:** ^1^Schulich School of Medicine and Dentistry, Western University, London, ON, Canada; ^2^Department of Paediatrics, Western University, London, ON, Canada; ^3^Children's Health Research Institute, London, ON, Canada; ^4^Department of Clinical Neurological Sciences, Western University, London, ON, Canada; ^5^Department of Epidemiology and Biostatistics, Western University, London, ON, Canada

**Keywords:** pediatric, critical care, trauma, brain injury, prognosis

## Abstract

**Aims and Objectives:** Severe traumatic brain injury (sTBI) is the leading cause of death in children. Our aim was to determine the mode of death for children who died with sTBI in a Pediatric Critical Care Unit (PCCU) and evaluate factors associated with mortality.

**Methods:** We performed a retrospective cohort study of all severely injured trauma patients (Injury Severity Score ≥ 12) with sTBI (Glasgow Coma Scale [GCS] ≤ 8 and Maximum Abbreviated Injury Scale ≥ 4) admitted to a Canadian PCCU (2000–2016). We analyzed mode of death, clinical factors, interventions, lab values within 24 h of admission (early) and pre-death (48 h prior to death), and reviewed meeting notes in patients who died in the PCCU.

**Results:** Of 195 included patients with sTBI, 55 (28%) died in the PCCU. Of these, 31 (56%) had a physiologic death (neurologic determination of death or cardiac arrest), while 24 (44%) had withdrawal of life-sustaining therapies (WLST). Median (IQR) times to death were 35.2 (11.8, 86.4) hours in the physiologic group and 79.5 (17.6, 231.3) hours in the WLST group (*p* = 0.08). The physiologic group had higher partial thromboplastin time (PTT) within 24 h of admission (*p* = 0.04) and lower albumin prior to death (*p* = 0.04).

**Conclusions:** Almost half of sTBI deaths in the PCCU were by WLST. There was a trend toward a longer time to death in these patients. We found few early and late (pre-death) factors associated with mode of death, namely higher PTT and lower albumin.

## Introduction

Severe traumatic brain injury (sTBI) is the leading cause of pediatric morbidity and mortality in high income countries (1, 2). TBIs are responsible for ~70% of traumatic deaths in the pediatric age group (2). In pediatric sTBI, outcome prediction is difficult, and outcomes vary between centers (3, 4). Retrospective cohort studies have explored the association of various factors in sTBI at hospital admission with outcomes (5–8). In these studies, however, mode of death is not reported. Of the patients who died, it is unknown how many died from cardiac or neurologic determination of death (NDD) (physiologic death) and how many died by withdrawal of life-sustaining therapies (WLST). Moreover, specific investigation of late (i.e., within 48 h preceding death) clinical and physiological factors associated with mortality in pediatric sTBI is lacking. How end-of-life treatment decisions are made by families and care providers is unknown.

Strong early predictors for mortality from sTBI have been identified at hospital admission and include: abnormal pupillary response, Injury Severity Score (ISS) >25, Glasgow Coma Scale (GSC) score, total duration of intracranial pressure (ICP) elevation >60 mmHg, the presence of intracranial bleeding (7), missing motor response or fixed and bilateral dilated pupils (9), mechanism of injury (8), subarachnoid hemorrhage (10), Rotterdam CT score (11), acquired hypernatremia (12), and early presentation of central diabetes insipidus (CDI) (5).

Mode of death has been studied in non-TBI pediatric populations, with a range of 40–70% of deaths by WLST (13–17). WLST decision-making has been explored in cohorts of children following cardiac arrest (13), and in the general pediatric critical care unit (PCCU) population (17). There exists considerable variability in neuroprognostication and its association with WLST within European PCCUs (16).

Our three objectives for this study were to (i) determine the mode of death for children who died following sTBI, (ii) examine which factors, both early and late, were associated with various modes of death, and (iii) explore the decision-making process leading to WLST in order to shape in-hospital family support structures and provide essential prognostic information to families when making end-of-life decisions.

## Methods

### Study Design and Participants

This retrospective cohort study included all severely injured (ISS ≥ 12) pediatric patients (>1 month and <18 years) admitted to the PCCU at the Children's Hospital, London Health Sciences Centre (LHSC) between January 1st, 2000 and December 31^st^, 2016 who died during their index PCCU admission. The Children's Hospital is the regional pediatric level I trauma centre for Southwestern Ontario, serving a geographical area of 19,000 km^2^ with a pediatric population over 400,000. sTBI was defined as a pre-sedation GCS score of 蠄 8, and a head Maximum Abbreviated Injury Scale (MAIS) of 蠅 4 (18). Patients were identified retrospectively from written and electronic admission records of the PCCU and cross-referenced with hospital records data and the provincial trauma registry to ensure that all eligible patients were captured (5, 12, 19). Approval was obtained from the Research Ethics Board at Western University.

The primary outcome was mode of death, which was classified as either physiological death (NDD or cardiac arrest) or WLST. NDD was defined by two national consensus statements, first published in 1999 and applied to patients admitted 2000–2006, and then updated in 2006 and applied for all subsequent patients (20, 21). We also reviewed whether Cerebral Blood Flow testing had been performed. Secondary outcomes included the number of family and multi-disciplinary meetings, meeting attendees, and timing of death.

**Figure 1:**
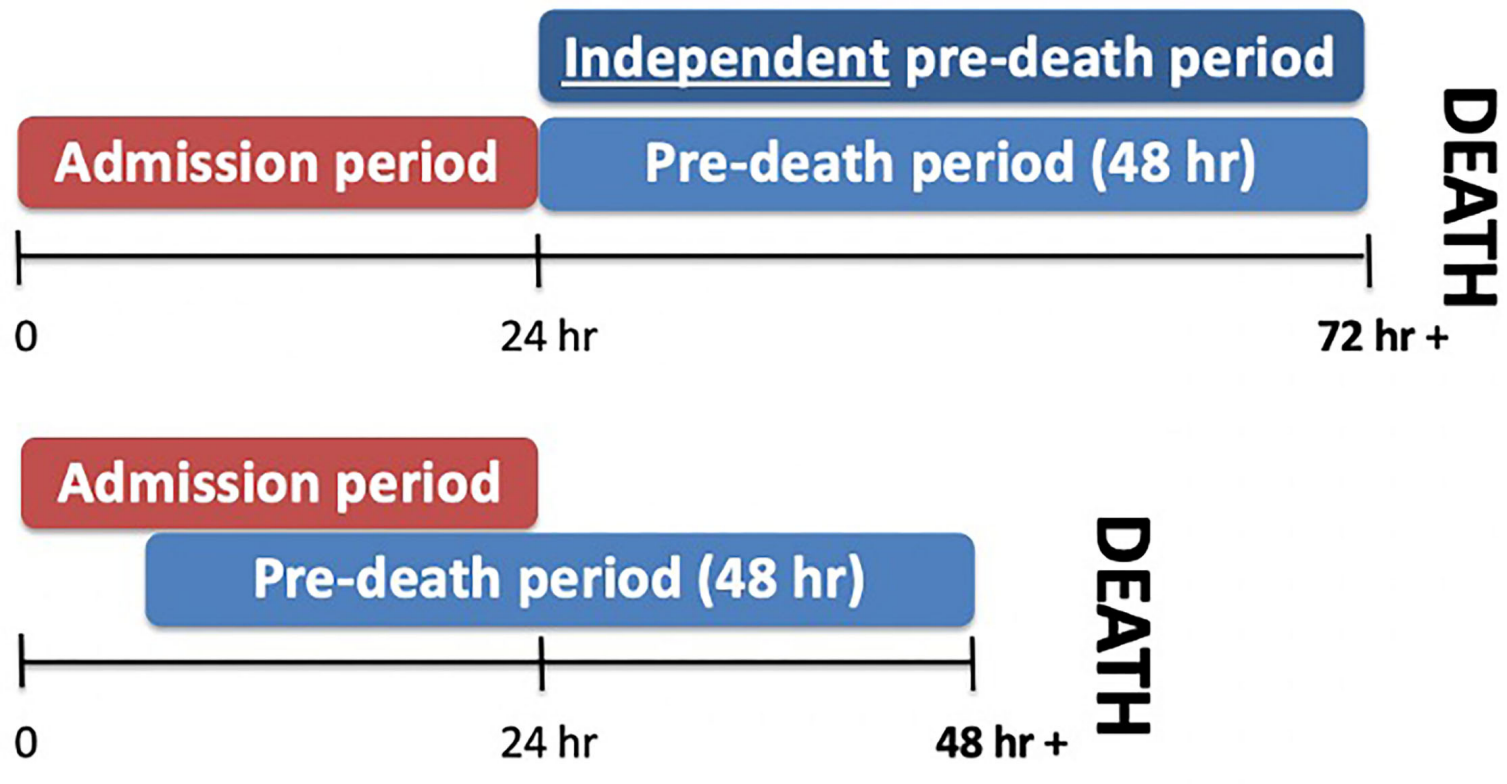
Time period definitions. Three time periods were defined. Admission values were recorded within 24 h of admission, while pre-death values were recorded within 48 h of death. Non-overlapping pre-death values were defined as independent pre-death values.

The electronic charting system, the paper copy of patients' hospital charts and the trauma registry were used to obtain the following data: age, sex, mechanism of injury, Injury Severity Score (ISS) at admission, GCS (pre-sedation, recorded prior to intubation either at the scene or referring hospital, or on arrival to LHSC), Rotterdam CT score, laboratory and neuroimaging results, interventions and outcomes. Laboratory and additional clinical variables were recorded over three time periods: admission (within 24 h of admission), pre-death (within 48 h of death) and independent pre-death (pre-death values in patients who died 72 h or more from admission, allowing for no overlap in admission and pre-death variables), constituting the worst values within 48 h prior to death (Figure 1). Laboratory variables included hemoglobin level, platelet count, serum albumin, international normalized ratio (INR), partial thromboplastin time (PTT), serum glucose, and neutrophil: lymphocyte ratio (NLR). Clinical variables recorded included pupillary response, episodes of hypotension [defined as systolic blood pressure (SBP) <70 for infants, SBP <70 + (2 x age) for toddlers and children less 10 years old, and SBP <90 mm Hg for children ≥ 10 years old] (22) and hypoxemia (PO2 <65 mm Hg, admission arterial blood gas). Specific documented abnormalities included skull fractures, cerebral edema (focal and diffuse), diffuse axonal injury (DAI), subarachnoid hemorrhage (SAH), subdural hemorrhage (SDH), intracranial hemorrhage (ICH), brain herniation, midline shift, cerebral contusion, and ischemia. Intervention variables included placement of an intracranial pressure (ICP) monitoring device, decompressive craniectomy, treatment with 3% hypertonic saline, mannitol, thiopental or barbiturate infusion, therapeutic hypothermia, desamino-8-D-arginine (DDAVP) and/or vasopressin, and transfusion of packed red blood cells (PRBC) or other blood products (5). Data on the number of family and multidisciplinary meetings and meeting attendees were collected along with the primary reason for the decision to withdraw life-support when applicable (prognosis, or prognosis and failure to meet brain death criteria).

### Statistical Methods

Continuous variables were summarized using medians (IQRs), and groups were compared using Mann-Whitney *U*-tests. Categorical variables were summarized using frequencies (%), and groups were compared using chi-square tests or Fisher's exact chi-square when appropriate. Poisson regression models were used to compare groups for count variables. Because the duration of the study spanned 16 years, we analyzed the data in specific time periods. The TBI guidelines were released in Pediatric Critical Care Medicine journal in 3 iterations: 2003, 2012, and 2016. We thus examined our primary outcome by time periods (2000–2003, 2004–2012, and 2012–2016). We also examined our primary outcome by the two NDD guidelines periods (2000–2006 and 2006–2016). All analyses were conducted with SPSS v.25 (IBM Corp., Armonk, NY, USA), and *p* < 0.05 were considered statistically significant.

## Results

We identified 195 patients with sTBI. Patient and injury demographics are reported in Table 1. The majority of the patients were male (*n* = 37, 67%). Forty percent (*n* = 126) had an ICP monitor placed. PCCU mortality was 28% (*n* = 55), and of these, 31 (56%) patients had a physiologic death. Further, the majority of these patients (*n* = 27, 84%) died by NDD, whereas five (16%) patients experienced cardiac death. The primary outcome was not associated with year of study or with NDD or TBI guideline update periods. Patients who died had lower pre-sedation GCS (*p* < 0.01), higher MAIS head (*p* = 0.047), higher MAIS thorax (*p* = 0.029), higher Rotterdam CT score (*p* < 0.01), and were more likely to have abnormal laboratory investigations, fixed pupils (*p* < 0.01) and be hypotensive (*p* < 0.01) within the admission period (Table 1). Twelve (22%) patients had an ICP monitor placed, 11 (20%) underwent decompressive craniectomy, 22 (40%) received hypertonic saline, and 33 (60%) received mannitol (Table 2).

**Table 1:** Bivariate analyses of admission variables, including demographic characteristics, laboratory data and CT findings of patients who died and survived in the PCCU.

**Variable**	**PCCU mortality (*n* = 55)**	**PCCU survival (*n* = 140)**	***P*-value**
Age, y	14 (1, 17)	12 (6, 16)	0.665
Male	37 (67.3)	94 (67.1)	0.986
Pre-sedation GCS	3 (3, 4)	6 (4, 7)	**<0.001**
ISS	33 (27, 43)	30 (26, 38)	**0.009**
Injury profile			
MAIS head	5 (5, 5)	5 (5, 5)	**0.047**
MAIS face	1 (1, 2)	1 (1, 2)	0.308
MAIS neck	1 (1, 1)	3 (1, 3)	0.248
MAIS thorax	3 (2, 3)	3 (2, 3)	**0.029**
MAIS abdomen	2 (2, 2)	2 (2, 3)	0.204
MAIS spine	2 (2, 3)	2 (1.5, 2)	**0.020**
MAIS extremities	2 (2, 3)	2 (2, 3)	0.349
MAIS external	1 (1, 1)	1 (1, 1)	0.573
Mechanism of injury			0.141
MVC	38 (69)	99 (70.7)	0.823
Fall	2 (3.6)	15 (10.7)	0.160
Intentional	10 (18.2)	12 (8.6)	0.056
Other	5 (9.1)	14 (10.0)	0.847
Rotterdam CT score	3 (3, 5)	2 (2, 3)	**<0.001**
CT characteristics			
SAH	29 (54.7)	53 (39.0)	**0.050**
IVH	17 (32.1)	26 (19.1)	0.056
Absent basal cisterns	24 (45.3)	8 (5.8)	**<0.001**
Compressed basal cisterns	7 (13.2)	6 (4.3)	**0.049**
Epidural Mass Lesion	50 (90.9)	121 (86.4)	0.391
Midline Shift >5 mm	9 (17.0)	13 (9.4)	0.143
Laboratory values			
Platelets,^*^10^9^/L	138.0 (85.0, 249.0)	198.5 (151.0, 254.8)	**0.001**
NLR^a^	6.5 (2.2, 13.5)	10.4 (5.2, 17.0)	**0.010**
INR, [PTt/PTn]^ISI^	1.5 (1.0, 2.0)	1.0 (1.0, 1.2)	**<0.001**
PTT, s	42 (32, 68)	31 (28, 36)	**<0.001**
Glucose, mmol/L^a^	10.4 (7.7, 14.1)	7.8 (6.8, 9.6)	**<0.001**
Hemoglobin, g/L	106 (83,125)	99 (84, 119.8)	0.507
Albumin, g/L	26 (19.5, 34.5)	36 (30, 39)	<0.001
Fixed pupils (*yes)	18 (32.7)	120 (85.7)	**<0.001**
Hypotension	24 (43.6)	22 (15.7)	**<0.001**

*Continuous variables are presented as median (IQR) and categorical variables as n (%). GCS, Glasgow Coma Scale; ISS, Injury Severity Score; MAIS, Maximum Abbreviated Injury Scale; SAH, subarachnoid hemorrhage; IVH, Intraventricular hemorrhage; NLR, neutrophil to lymphocyte ratio; INR, International Normalized Ratio; PTT, partial thromboplastin time*.

a *Highest value in first 24 h (all other values are taken from admission blood draw). p values < 0.05 were bolded*.

**Table 2:** PCCU interventions for the management of sTBI by mode of death.

**PCCU intervention**	**Physiologic death (*n* = 31)**	**WLST (*n* = 24)**	***P*-value**
Placement of ICP monitoring device	5.00 (16.1)	7.00 (33.3)	0.188
Decompressive craniectomy	5.00 (16.1)	6.00 (28.6)	0.318
Treatment with 3% HS	11.0 (35.5)	11.0 (52.4)	0.226
Treatment with mannitol	16.0 (51.6)	17.0 (81.0)	**0.031**
Barbiturate infusion	5.00 (16.1)	5.00 (23.8)	0.500
Thiopental infusion	7.00 (22.6)	6.00 (28.6)	0.624
Therapeutic hypothermia	2.00 (6.50)	0 (0)	0.509
DDAVP +/- vasopressin	22.0 (71.0)	14.0 (66.7)	0.742
PRBC transfusion	25.0 (80.6)	19.0 (90.5)	0.449
Other blood product transfusion	23.0 (74.2)	11.0 (52.4)	0.105

*Data are presented as n (%). ICP, intracranial pressure; PRBC, packed red blood cells. p values < 0.05 were bolded*.

### Time to Death

Children who died by WLST had a trend to a greater time to death with a median (IQR) value of 79.5 (17.6, 231.3) hours compared to children who had a physiologic death with a median (IQR) value of 35.2 hours (11.8, 86.4) (*p* = 0.08).

### Clinical, Interventional and Laboratory Predictors of Mode of Death

Children who died by a physiologic death were similar to children who died by WLST for most factors (Tables 2, 3). A greater number of children who died by WLST received mannitol (*p* = 0.03) (Table 2) but this was no longer significant when controlling for time to death. Children who had a physiologic death had higher PTT values within the admission period and lower albumin (*p* = 0.035) in the independent pre-death period, after controlling for potential confounders.

**Table 3:** Clinical and laboratory findings on admission and prior to death by mode of death.

**Variables**	**Physiologic death (*n* = 31)**	**WLST (*n* = 24)**	***P*-value**
**Admission**			
Age, y	15.0 (5.00, 17.0)	13.5 (1.00, 17.00)	0.499
GCS	3.00 (3.00, 4.00)	3.00 (3.00, 4.00)	0.655
ISS	33.0 (27.0, 43.0)	32.5 (27.0, 41.8)	0.670
Rotterdam CT score	3.00 (3.00, 5.00)	4.00 (3.00, 5.00)	0.400
Fixed pupils	10.0 (32.3%)	8.00 (33.3%)	0.933
Hypotension	13.0 (41.9%)	11.0 (45.8%)	0.773
Hemoglobin, g/L	106 (83.0,124)	111(83.8,130)	0.581
Platelets, *10^9^/L	134 (61.0, 217)	151 (93.0, 273)	0.219
Albumin, g/L	25.0 (16.0, 32.3)	30.0 (23.0, 39.0)	0.055
INR, [PTt/PTn]^ISI^	2.00 (1.00, 3.00)	1.20 (1.00, 2.00)	0.216
PTT, s	48.0 (36.0, 83.0)	37.0 (29.0, 53.0)	**0.037**
Glucose, mmol/L	9.90 (6.70, 14.0)	10.5 (7.78, 16.6)	0.652
NLR	5.375 (2.24, 12.9)	6.916 (2.57, 16.8)	0.575
**Pre-death**			
Fixed pupils	17.00 (89.5%)	12.00 (85.7%)	1.000
Hypotension	7.00 (38.9%)	2.00 (14.3%)	0.235
Hemoglobin, g/L	81.0 (73.0, 95.0)	85.0 (78.0, 95.0)	0.445
Platelets, *10^9^/L	167 (94.0, 237)	169 (116, 235)	0.768
Albumin, g/L	23.0 (17.0, 31.0)	29.0 (22.0, 32.0)	0.186
INR, [PTt/PTn]^ISI^	1.60 (1.20, 2.20)	1.50 (1.30, 1.68)	0.526
PTT, s	44 (32.0, 71.0)	38.0 (30.5, 41.5)	0.215
Glucose, mmol/L	14.0 (8.50, 20.9)	8.30 (7.15, 15.9)	0.074
NLR	10.915 (5.23, 17.6)	8.615 (6.89, 10.14)	0.537
**Independent pre-death**			
Fixed pupils	6.00 (75.0%)	8.00 (80.0%)	1.000
Hypotension	2.00 (25.0%)	0.00 (0%)	0.183
Hemoglobin, g/L	85.0 (75.8, 98.0)	93.0 (81.8, 102)	0.477
Platelets, *10^9^/L	158 (94.5, 203)	173.5 (130, 276)	0.424
Albumin, g/L	18.5 (15.8, 24.8)	29.0 (23.0, 30.5)	**0.035**
INR, [PTt/PTn]^ISI^	1.55 (1.23, 2.80)	1.40 (1.30, 1.50)	0.413
PTT, s	36.5 (32.0, 67.8)	34.0 (30.0, 42.0)	0.295
Glucose, mmol/L	8.50 (7.95, 18.6)	7.50 (7.00, 8.80)	0.098
NLR	11.045 (5.60, 17.8)	8.67 (7.23, 11.0)	0.537

*Continuous variables are presented as median (IQR) and categorical variables as n (%). GCS, Glasgow coma scale; ISS, injury severity score; NLR, neutrophil to lymphocyte ratio; INR, international normalized ratio; PTT, partial thromboplastin time. p values < 0.05 were bolded*.

### Family and Multidisciplinary Meetings

There was no significant association between number of formal, informal or total family meetings and mode of death (Table 4). A median (IQR) of 2.0 (1.0, 4.0) documented family meetings occurred in all children who died from sTBI in the PCCU. Children's Aid Society (the regional child protection agency) or police were involved during the family meetings of four patients, whereas religious figures were present during a meeting for three patients. Pastoral support was noted to be included in meetings for five (10%) families. The median (IQR) for maximum number of attendees at formal family meetings was 3.0 (2.0, 5). Multidisciplinary meetings were documented in three of the 51 patient records.

**Table 4:** Family meetings.

	**WLST**	**Physiologic**	***P*-value**
Family meetings total	2.00 (1.00, 4.00)	2.00 (1.00, 4.00)	0.173
Formal family meetings	1.00 (1.00, 4.00)	1.00 (0.00, 2.00)	0.333
Informal family meeting	0.00 (0.00, 1.00)	1.00 (0.00, 1.00)	0.323

### Reason for WLST

Access to the paper chart with complete family meeting data was available in 21 (91%) patients who died by WLST. Family meeting prior to WLST was documented in the remaining 3 patients with limited detail in online death summaries. Documentation of family meetings in those with complete data showed that 14 (66%) patient families expressed poor prognosis as the key reason for pursuing WLST, while 5 (24%) added “failure to meet brain death criteria.” Some common documented expressions included: “Low likelihood of meaningful recovery,” and “do not want their child to suffer further” or “they would not want to live this way.” Of the 24 patients who died by WLST, evaluation of cerebral blood flow (a nucleotide scan or magnetic resonance angiography) was completed for 13 (54%) patients.

## Discussion

Understanding the mode and predictors of mortality in sTBI is crucial to our management of patients with sTBI. To our knowledge, this is the first study to demonstrate that children with sTBI died by WLST almost as frequently as by physiologic means. There was a trend toward a doubling in PCCU length of stay (LOS) in those who died by WLST, and year of study and updates in guidelines were not associated with mode of death. We also showed that most of the patients' clinical findings on admission and prior to death were similar in both groups. Documentation of family meetings prior to WLST was available in all cases, and there was a common language used to describe reasons for WLST.

Our rate of WLST mirrors those published in both American (14) and Canadian (15) studies of all inpatient pediatric deaths, with rates ~40–45%. The most common underlying diagnosis in these earlier studies was cardiovascular, with prematurity and neurological disease among the top four. Mode of death may be different post cardiac arrest. By applying our mode of death definition to a study population of cardiac arrest victims from the Netherlands, over 2/3 of the participants had WLST (13). The heavier emphasis on WLST in this Dutch population may be a result of fewer cardiac arrest early-survivors dying by NDD. Only 29% of this cohort met NDD criteria, compared to 49% in our sTBI cohort.

Not surprisingly, there was a trend to a longer time to death in the WLST group compared to the physiologic death group. A 10-year review of mortality in a UK PCCU found that median LOS in children who died varied according to mode of death with WLST of 3 days, and 1 day for brain death or failed CPR (23). This finding may be explained by uncertainty about prognosis. Prognostic accuracy may improve with time, as described in pediatric cardiac arrest early-survivors (24). In addition, there may be decision-maker conflict. Physicians are obligated to support the parental decision-making process and ensure care plans are consistent with the families' goals (25). Finally, there may be a desire to extend life-sustaining therapy to allow for family visitation or religious rites.

NDD criteria were updated in Canada in 2006 (26), while international pediatric TBI guideline updates occurred in 2003 and 2012 (27, 28). Our analysis, however, suggests these updates did not impact mode of death in children. Our results likely reflect the lack of major practice changes in the guideline updates; however, there are a number of potential barriers that lead to delays in guideline implementation, including lack of physician awareness or agreement. Additionally, limitations in applicability (e.g., due to complexity) could also play a role (29). In the context of pediatric TBI guidelines, cost has not been shown to be a factor in guideline adoption (30).

The recommendation to use invasive ICP monitoring for patients with severe TBI was another relevant guideline that did not appear to universally impact the study patients. There is significant variability in ICP monitoring practices, from 8 to 59% reported of a similar timeframe (31, 32). One single centre Canadian study evaluated reasons for not pursuing ICP monitoring, and found the main reasons were improving GCS, moribund status, and decision for clinical surveillance (33). We do not have data on decision for not pursuing ICP monitoring at our centre, but likely moribund status and opting for clinical surveillance prevailed. In these scenarios, ICP-targeted therapies would have been instituted in the setting of clinical deterioration.

Our results affirmed previously established strong predictors of pediatric sTBI mortality at the point of admission (Table 1). These include abnormal pupillary response, ISS > 25, GCS score, the presence of intracranial bleeding (7), missing motor response or fixed and bilateral dilated pupils (9), mechanism of injury (8), subarachnoid hemorrhage (10), Rotterdam CT score (11), acquired hypernatremia (12), and early presentation of central diabetes insipidus (CDI) (5, 34). While previous investigations have evaluated associations of admission values with outcomes (5–8), this study is significant in its consideration of pre-death variables and mode of death. Admission and pre-death values in this study were often close in time and even overlapped in 37 (67%) patients and resulted in limited detection of differences between these two time periods.

We found that higher PTT and lower albumin were associated with physiologic death in the admission and pre-death periods, respectively. Greater volume resuscitation in this group may help explain this finding. Volume resuscitation has been shown to drop albumin by 20% (35). Our findings may also be explained by the established association of focal brain injury and hepatocellular damage (36). This occurs via a rapid increase in chemokine expression that can further amplify the local brain injury response (37). The relatively lower hepatic function in the physiologic death group (as reflected in a higher PTT and lower albumin) may represent a more significant acute phase response and associated amplification of local brain injury responses. Moreover, the abnormal albumin in the physiologic group may be explained by its established identity as a negative acute phase reactant (38). The association of increased PTT with physiologic death may also be considered representative of trauma-induced coagulopathy, occurring independently of general hepatic dysfunction. Abnormalities in laboratory markers of coagulation such as PTT have been associated with mortality (9, 10, 38). Moreover, early coagulopathy may be an independent predictor of mortality in children after severe trauma (39).

The paucity of significant differences between our WLST and physiologic death groups may be explained simply by the fact that they were more similar than different. We surmise that those children who died physiologically were worse on the spectrum of injury severity, in part due to their trend to a shorter time to death. The majority of the physiologic group died by NDD. Patients who die by WLST in the setting of severe brain damage often meet many criteria for NDD (e.g., fixed pupils) but not all criteria (e.g., negative apnea test). The fact that 54% of WLST had cerebral blood flow testing suggests ~$\frac{1}{2}$ of WLST patients had near complete NDD testing, requiring the additional cerebral blood flow testing to distinguish NDD from near-NDD. Our findings suggest that it is difficult to prognosticate between those patients who die by NDD to those who die otherwise (e.g., die by WLST). This warrants further exploration with a larger sample in a multi-centre setting.

Though no trends were seen in the number of family meetings and outcomes, Children's Aid Society or police involvement was found to be associated with a slightly greater number of family meetings (median 2.5 vs. 2). This finding reflects the added complexity when non-accidental injury is considered. One important finding was the poor documentation of multi-disciplinary meetings, both in quality and in frequency. Accurate documentation is important for continuity of care, family support, and case reviews. Electronic medical records and physician reimbursement schemes supported by documentation should enable improved documentation.

The content of the family meetings provided insight into the decision-making process preceding WLST. Over 90% of families were documented as citing the child's poor prognosis as their primary reason for WLST in this study. Failure to meet brain death criteria in the setting of poor prognosis was expressed by others. A single-centre Norwegian study exploring WLST decision-making in adult sTBI found that futility was the rationale cited in a majority of cases (40). WLST decision-making has also been explored in cohorts of children admitted to the PCCU and following cardiac arrest. Fontana et al. (17) found that chronic illness and patient dying despite intervention were the most frequently documented contributors to WLST decision-making for children admitted to the PCCU. Hunfeld et al. (16) found considerable variability in neuroprognostication and its association with WLST between European PCCUs, following cardiac arrest. They reported that once neurological prognosis was determined to be futile, only 55% of centres considered pursuing WLST (41).

Our study has several limitations. First, this was a single centre, retrospective study. As well, the study did not include ICP values and, mortality during PCCU stay, but not after discharge from the PCCU, was captured by our database. Longer-term mortality is an important area of future research. Notably, our findings were strengthened by our clear variable definitions and regular quality checks. A future prospective study would allow for more uniform data collection. Future qualitative analysis of family meetings to understand their significance and factors contributing to the decision to WLST is worthwhile.

## Conclusions

Almost half of sTBI deaths in the PCCU were by WLST; there was a trend toward a longer time to death in these patients. Both groups were very similar, contrary to what one would expect, reflecting similar pathophysiological processes. This study introduces the idea of distinct predictive characteristics of mortality in sTBI associated with WLST and physiologic death. However, only a few early and late factors were identified with mode of death, namely higher PTT and lower albumin. Further investigation can help our understanding of predictors of mode of death and how decisions are made for end-of-life, which are important in how we care for sTBI patients and their families.

## Data Availability Statement

The raw data supporting the conclusions of this article will be made available by the authors, without undue reservation.

## Ethics Statement

The studies involving human participants were reviewed and approved by Western University Research Ethics Board. Written informed consent from the participants' legal guardian/next of kin was not required to participate in this study in accordance with the national legislation and the institutional requirements.

## Author Contributions

This study was conceived by DF and JT and completed under their supervision. TB conducted the qualitative data collection and part of the quantitative data collection. Earlier data collection, essential in forming the study database was conducted by KA and TC. Data analysis was performed by MM. This manuscript was written by TB and JT reviewed and edited by the remaining author group. All authors contributed to the article and approved the submitted version.

## Funding

This study was funded by the Children's Health Research Institute (CHRI) in London, Ontario through their CHRI Internal Research Grant Fund.

## Conflict of Interest

The authors declare that the research was conducted in the absence of any commercial or financial relationships that could be construed as a potential conflict of interest.

## Publisher's Note

All claims expressed in this article are solely those of the authors and do not necessarily represent those of their affiliated organizations, or those of the publisher, the editors and the reviewers. Any product that may be evaluated in this article, or claim that may be made by its manufacturer, is not guaranteed or endorsed by the publisher.
